# A Prospective Open‐Label Observational Study of a Buffered Soluble 70 mg Alendronate Effervescent Tablet on Upper Gastrointestinal Safety and Medication Errors: The GastroPASS Study

**DOI:** 10.1002/jbm4.10510

**Published:** 2021-05-17

**Authors:** Salvatore Minisola, Antonio P Vargas, Giulia Letizia Mauro, Fernando Bonet Madurga, Giovanni Adami, Dennis M Black, Nawab Qizilbash, Josep Blanch‐Rubió

**Affiliations:** ^1^ Department of Clinical, Internal, Anaesthesiology, and Cardiovascular Sciences Sapienza University of Rome Rome Italy; ^2^ SEPAR, S.L. (Private Clinic), Calle Linaje Malaga Spain; ^3^ Department of Surgical, Oncologic, and Stomatologic Disciplines University of Palermo Palermo Italy; ^4^ MDS 360 (Private Clinic), Chamartín Madrid Spain; ^5^ Division of Rheumatology, Department of Medicine University and Azienda Ospedaliera Universitaria Integrata of Verona Verona Italy; ^6^ University of California San Francisco CA USA; ^7^ Epidemiology and Risk Management, Oxon Epidemiology Madrid Spain; ^8^ Faculty of Epidemiology and Population Health London School of Hygiene and Tropical Medicine London UK; ^9^ Rheumatology Service Hospital del Mar, Passeig Marítim and IMIM (Hospital del Mar Medical Research Institute), Parc de Recerca Biomèdica de Barcelona Barcelona Spain

**Keywords:** ALENDRONATE, EFFERVESCENT, GASTROINTESTINAL ADVERSE EVENTS, OSTEOPOROSIS, POSTMENOPAUSAL WOMEN

## Abstract

Upper gastrointestinal (GI) side effects are a main reason for discontinuing bisphosphonate treatment, an important therapeutic option for osteoporosis patients. Consequently, the development of novel formulations with improved tolerability is warranted. In this multicenter prospective, observational, postauthorization safety study conducted in Italy and Spain, postmenopausal women (PMW) with osteoporosis (naïve to bisphosphonates) were treated weekly with a buffered soluble alendronate 70 mg effervescent (ALN‐EFF) tablet (Binosto®) and followed for 12 ± 3 months. Information was collected on adverse events (AEs), medication errors, persistence, and compliance using the Morisky‐Green questionnaire. Patients (*N* = 1028) aged 67 ± 9 years (mean ± SD) received ALN‐EFF weekly. The cumulative incidence of upper GI AEs (oesophageal toxicity, gastritis, gastric ulcers, and duodenitis) related to ALN‐EFF (primary endpoint) was 9.6% (95% confidence interval [CI] 7.9–11.6%), the vast majority being of mild intensity. The most frequently occurring upper GI AEs related to ALN‐EFF were dyspepsia (2.7%), gastroesophageal reflux disease (2.4%), and nausea (2.2%). None of the relevant upper GI AEs listed in the primary endpoint and no serious AEs were reported. At least one medication error occurred in 29.9% (95% CI 27.1–32.8%) of patients. However, the majority of medication errors were associated with administration instructions applicable to any oral bisphosphonate and only seven medication errors were associated with the ALN‐EFF formulation. ALN‐EFF was discontinued in 209 of 1028 (20.3%) patients. The most frequent reasons for discontinuation were AEs related to ALN‐EFF (46.9%) and patients' decision (42.6%). Compliance with ALN‐EFF was high, reflected by a mean Morisky‐Green score of 92.8 ± 18.6. PMW with osteoporosis treated with ALN‐EFF in a real‐world setting experienced few upper GI AEs. In addition, they had a low discontinuation and high compliance compared with other formulations, suggesting that ALN‐EFF may increase patient satisfaction and therefore long‐term adherence and efficacy. © 2021 The Authors. *JBMR Plus* published by Wiley Periodicals LLC on behalf of American Society for Bone and Mineral Research.

## 1 Introduction

Osteoporosis is characterized by abnormalities in bone mass and structure in bone tissue leading to impaired skeletal strength and increased susceptibility to fractures.^(^ ^1^ ^)^ Osteoporotic fractures are a major cause of morbidity, decreased quality of life, and increased mortality worldwide.^(^ ^2^, ^3^, ^4^ ^)^

For postmenopausal women (PMW), antiresorptive agents are usually the first‐line treatment and include bisphosphonates (alendronate, risedronate, zoledronate, or ibandronate) or denosumab.^(^ ^5^, ^6^ ^)^ Alendronate 70 mg tablet once weekly is the most commonly used bisphosphonate. Although alendronate has been shown to reduce the risk of vertebral, non‐vertebral, and hip fractures by 55%, 64%, and 47%, respectively,^(^ ^7^ ^)^ adherence is problematic, with >50% discontinuing treatment within the first year.^(^ ^8^, ^9^ ^)^

Upper gastrointestinal (GI) side effects are one of the main reasons for discontinuing bisphosphonate treatment, observed in both real‐life studies^(^ ^10^, ^11^, ^12^ ^)^ and clinical trials,^(^ ^13^, ^14^, ^15^, ^16^ ^)^ resulting in reduced anti‐fracture efficacy and increased health care costs.^(^ ^17^, ^18^, ^19^, ^20^ ^)^

The International Osteoporosis Foundation and the European Calcified Tissue Society recognize the challenges with adherence associated with oral bisphosphonates.^(^ ^21^ ^)^

In randomized clinical trials, the incidence of upper GI side effects in PMW treated with standard alendronate tablets (standard formulation) range from 20% to 30%, with similar incidence obtained for comparator groups in these trials.^(^ ^13^, ^14^, ^15^, ^16^ ^)^ These values remain high, necessitating alternative therapeutic approaches.

To address this, Binosto®, a novel buffered soluble alendronate 70 mg effervescent tablet formulation (ALN‐EFF), was specifically developed to improve GI tolerability through its effervescent formulation that has strong buffering properties.^(^ ^22^, ^23^ ^)^ It provides a more convenient formulation to facilitate compliance, particularly in patients having difficulty swallowing tablets. ALN‐EFF is ingested as a buffered solution of fully dissolved alendronate to prevent the pH of gastric juice from further decreasing in the presence of alendronate, diminishing potential damage in cases of esophageal reflux and preventing contact of solid alendronate with the esophageal mucosa.^(^ ^5^ ^)^

It has to be taken at least 30 minutes before the first meal of the day to permit adequate absorption. The effervescent tablet should be fully dissolved in plain water (≥120 mL) before consumption. This is to minimize esophageal irritation and esophageal toxicity as well as interactions with food items that limit the absorption of alendronate. ALN‐EFF is bioequivalent to Fosamax and was approved as a hybrid medicine in 2011 in the European Union and by the US Food and Drug Administration (FDA) in 2012.^(^ ^23^, ^24^ ^)^

The aim of this study was to investigate the incidence of upper GI adverse events (AEs; (esophageal toxicity, gastritis, gastric ulcers, and duodenitis) and medication errors associated with ALN‐EFF.

## 2 Subjects and Methods

### 2.1 Study design

This was a prospective, observational, multicenter, multinational, single‐arm, postauthorization safety study (PASS) to evaluate the incidence of upper GI AEs and medication errors during 12 months of treatment with ALN‐EFF. This study was registered on The European Union electronic Register of Post‐Authorisation Studies (EU PAS Register): ENCEPP/SDPP/10888. Patients were enrolled from primary and secondary care centers in Spain (16 centers) and Italy (7 centers) from May 2017 to February 2020.

The mean (±SD) follow‐up period was 12 ± 3 months with data collection time points at baseline, up to 2 months (“early” follow‐up), 2 to 9 months (“intermediate” follow‐up), and 9 to 15 months (“late” follow‐up) after baseline.

Patients were followed from the date of the first prescription of ALN‐EFF (defined as index date).

The occurrence of upper GI AEs (ie, including esophageal toxicity, gastritis, gastric ulcers, and duodenitis) was identified at routine visits during this period. Only AEs occurring within 12 ± 3 months of the index date were considered eligible.

At baseline, demographic data and levels of serum calcium were collected along with medical history about osteoporosis, fractures, and history of GI symptoms, as well as prior and concomitant treatment.

The study was designed, conducted, and reported in accordance with the ethical principles laid down in the Declaration of Helsinki, the Good Pharmacoepidemiology Practices guidelines of the International Society for Pharmacoepidemiology, and applicable local rules and regulations. The protocol was approved by the Medicines and Healthcare Regulatory Authority in the UK.

### 2.2 Patient selection and eligibility criteria

PMW were offered the possibility to participate in the study at the discretion of the treating physician only after the decision to prescribe ALN‐EFF for the treatment of osteoporosis was made. Patient selection was performed during routine clinical practice by the physician in accordance with ALN‐EFF product information.^(^ ^22^ ^)^

Inclusion criteria were patients newly prescribed ALN‐EFF and naïve to bisphosphonate therapy; women with osteoporosis; written informed consent; and the ability to comply with the requirements of the study. Patients with a history of upper GI symptoms were also included. Exclusion criteria were the presence of any contraindications to ALN‐EFF according to Summary of Product Characteristics (SmPC).^(^ ^22^, ^23^ ^)^

### 2.3 Study objectives

The primary objective of this study was to assess the cumulative incidence of upper GI AEs (esophageal toxicity, gastritis, gastric ulcers, and duodenitis) with ALN‐EFF, and the co‐primary objective was to assess the incidence of medication errors during treatment. Secondary aims were to evaluate persistence, discontinuation and reasons for discontinuation, and compliance with the SmPC recommendations for ALN‐EFF^(^ ^22^, ^23^ ^)^ and to calculate the incidence rate of ALN‐EFF individual gastric AEs. We also evaluated the association of a range of clinical characteristics (determinants) on the cumulative incidence of upper GI AEs with ALN‐EFF.

### 2.4 Outcome measures

During follow‐up visits, information on AEs, medication errors (capture potential errors in the method of administration via questionnaire; Supplemental Table Table S1), and treatment discontinuation were collected.

The following AEs were coded by MedDRA version 20.0^(^ ^25^ ^)^ using preferred terms (PTs) that encompassed the primary endpoint for analysis of the cumulative incidence of upper GI AEs: esophagitis, esophageal ulcer, esophageal perforation, esophageal bleeding (PT: esophageal hemorrhage), esophageal stricture (PT: esophageal stenosis), gastritis, gastric ulcer, gastric perforation, gastric bleeding (PT: gastric hemorrhage), gastric stricture (PT: gastric stenosis), duodenitis, duodenal ulcer, duodenal perforation, duodenal bleeding (PT: small intestinal hemorrhage), duodenal stricture (PT: duodenal stenosis), and melaena. The presence of serious AEs (defined as an adverse reaction that results in death, is life‐threatening, requires hospitalization or prolongation of existing hospitalization, results in persistent or significant disability or incapacity, or is a birth defect) were also recorded.

In addition, other PTs were also analyzed within the primary endpoint of upper GI AEs (defined above) as they were considered potentially consistent with esophageal toxicity, gastritis, gastric ulcers, and duodenitis. These included abdominal pain, upper abdominal pain, dyspepsia, gastroesophageal reflux disease, nausea, abdominal distension, dysphagia, Helicobacter gastritis, Helicobacter infection, vomiting, and esophageal pain. Compliance was assessed using the Morisky‐Green 4‐question scale^(^ ^26^ ^)^ and the number of tablets missed since the previous visit.

### 2.5 Statistical analysis

A total of 1200 PMW with osteoporosis were planned to be enrolled to achieve 1000 evaluable patients allowing a precision (95% confidence intervals CIs]) of ±1% if the observed incidence of upper GI AEs is approximately 2%.

Data are presented as mean ± standard deviation (SD) for continuous variables and number and % for categorical variables. The cumulative incidence (proportion) of primary and secondary endpoints was calculated along with respective 95% CIs. The proportion (and 95% CIs) of subjects with medication errors was calculated. Persistence/discontinuation was analyzed using Kaplan–Meier curves and life tables. Multivariate logistic regression models were used to explore predictor variables influencing the occurrence of upper GI AEs related to ALN‐EFF, expressed as odds ratios (OR) and respective 95% CIs. Compliance using the Morisky‐Green score and tablet count are reported as mean scores (out of 100) ± SD. Where comparisons were made, quoted *p* values are two‐tailed and a value of *p* < 0.05 was considered statistically significant. Missing values were described with no imputation. All analyses were performed using SAS version 9.4 and SAS Enterprise Guide, version 7.1 (SAS Institute, Cary, NC, USA).

## 3 Results

### 3.1 Patient disposition and baseline characteristics

**Fig 1:**
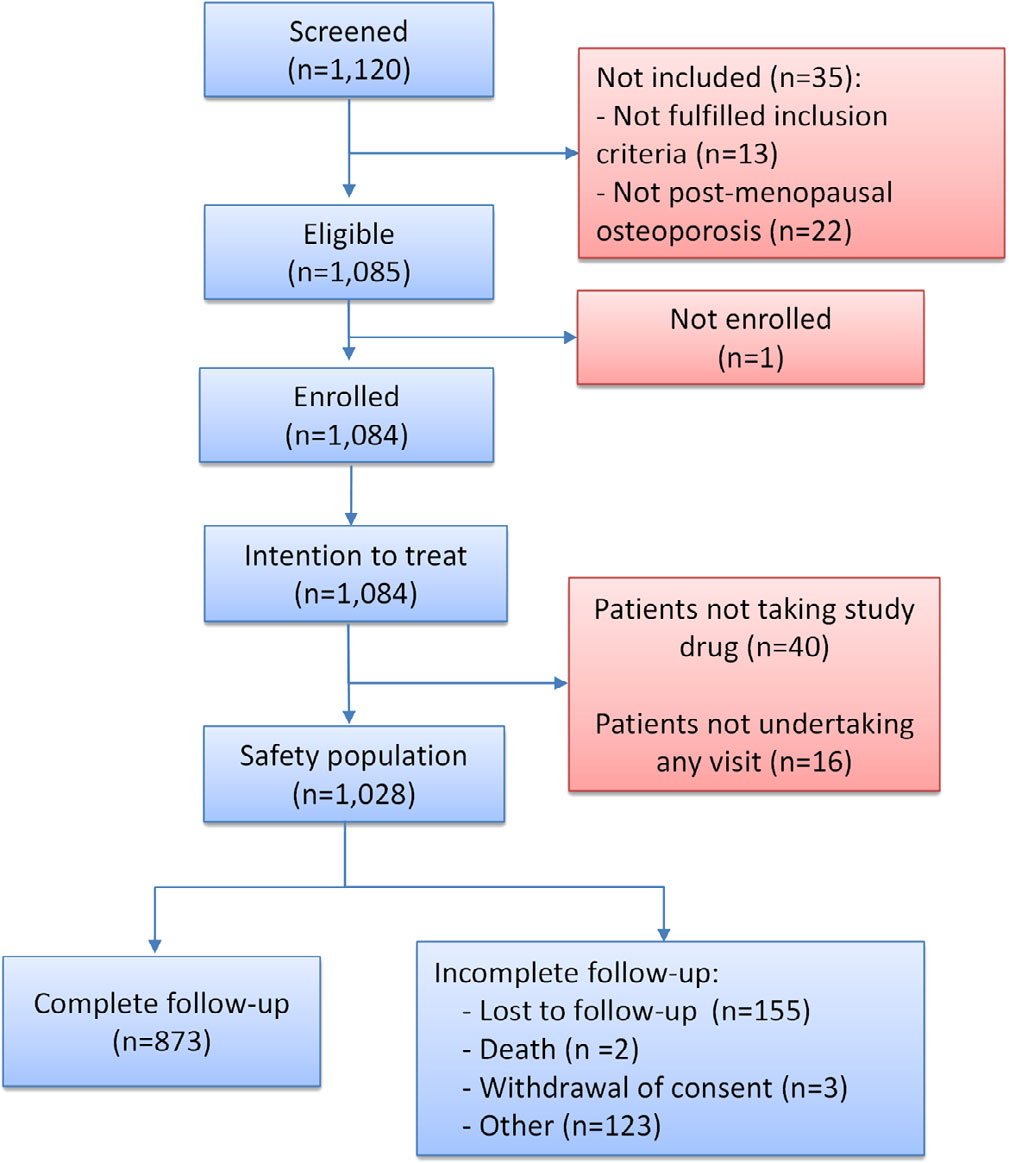
Patient disposition.

A total of 1120 patients were screened and 1085 were eligible to participate in the study. Thirty‐five were excluded (13 did not fulfill inclusion criteria for patients newly prescribed ALN‐EFF and naïve to bisphosphonate therapy and 22 patients were excluded due to off‐label use) and 1 patient was not enrolled. A further 40 patients were excluded from analysis after enrollment because they did not take the study drug and 16 did not perform any follow‐up visits. Of the 1028 evaluable patients (646 from 16 centers in Spain ranging from 1 to 89 patients and 382 from seven centers in Italy ranging from 3 to 146 patients), 873 completed the 12 ± 3‐month follow‐up visit, and 155 patients did not complete all follow‐up visits, 2 died, and 3 withdrew consent (Fig. 1). These 1028 patients represented the Safety Set considered in this analysis.

Baseline clinical characteristics for all 1028 PMW are summarized in Table 1. A total of 646 (62.8%) participated from 17 centers in Spain, and the remaining 382 (37.2%) from seven centers in Italy. Mean age was 67 ± 9 years and age at menopause was 49 ± 5 years. Just over one‐third (*n* = 370; 36%) of patients had a previous fracture, the most frequent being vertebral (*n* = 197; 53.2%) and 856 patients (83.3%) had at least one comorbid disease. 319 (31%) patients had a history of GI symptoms of which 271 (85%) were upper GI symptoms, the most frequent being dyspepsia (*n* = 108; 33.9%) and gastritis (*n* = 103; 32.3%). While all patients were naïve to bisphosphonates, 111 (10.8%) had previously received another treatment for osteoporosis (Table 2). The majority of patients enrolled in the study were receiving vitamin D and/or calcium supplementation (cholecalciferol [*n* = 545, 87.2%] and/or calcium [*n* = 260; 41.6%]) and 724 (70.4%) patients were receiving medication to treat other comorbidities. These were mainly antihypertensives (*n* = 402, 55.5%) anti‐inflammatories (*n* = 204, 28.2%), antidepressants (*n* = 171, 23.6%), statins (*n* = 169, 23.3%), and proton‐pump inhibitors (*n* = 95, 13.1%).

**Table 1:** Characteristics of the Study Population

Clinical characteristics	*n* (%)
No. of subjects	1028 (100)
Spain	646 (62.8)
Italy	382 (37.2)
Age (years), mean ± SD	67 ± 9
White race	1011 (98)
Body mass index (Kg/M^2^), mean ± SD	25.6 ± 4.3
Age at onset of menopause (years), mean ± SD	49 ± 5
Time since onset of menopause (years), mean ± SD	17.4 ± 9.9
Smoking habits (ex or current smoker)	264 (25.8)
Serum calcium (mmol/L), mean ± SD	2.4 ± 0.21
Prone to falling (yes)	99 (9.7)
No. of previous falls, mean ± SD	2.6 ± 2.6
Previous bone fracture	370 (36)
Vertebral	197 (53.2)
Wrist	69 (18.7)
Ribs	24 (6.5)
Other	108 (29.2)
Comorbid diseases	856 (83.3)
Ovariohysterectomy/untreated hypogonadism/early menopause	186 (21.7)
Thyroid/parathyroid disorder	154 (18)
Diabetes mellitus (type 1 or 2)	88 (10.3)
Rheumatoid arthritis	58 (6.8)
Chronic obstructive pulmonary disease	35 (4.1)
Other	745 (87)
Gastrointestinal symptoms	319 (31)
Upper gastrointestinal symptom	271 (85)
Type of gastrointestinal symptom	
Dyspepsia	108 (33.9)
Gastritis	103 (32.3)
Acid regurgitation	98 (30.7)
Constipation	71 (22.3)
Flatulence	70 (21.9)
Abdominal distension	56 (17.6)
Abdominal pain	27 (8.5)
Other	46 (14.4)

Data are presented as *n* (%) unless indicated otherwise.

**Table 2:** History of Osteoporosis Treatment, Supplementation, and Concomitant Medication

Treatment	*n* (%)
No. of subjects	1028 (100)
History of osteoporosis treatment	111 (10.8)
Denosumab	48 (43.2)
Teriparatide	23 (20.7)
Strontium ranelate	21 (18.9)
History of osteoporosis supplement	625 (60.8)
Cholecalciferol	545 (87.2)
Calcium	260 (41.6)
Other	47 (18.1)
History of concomitant medication	724 (70.4)
Antihypertensive	402 (55.5)
Anti‐inflammatory (for rheumatoid/osteoarthritis)	204 (28.2)
Antidepressant	171 (23.6)
Statin	169 (23.3)
Proton pump inhibitor	95 (13.1)
Antidiabetic	81 (11.2)
Reason for starting ALN‐EFF	
New osteoporotic patient	916 (89.1)
Problems with previous medications^a^	112 (10.9)
Patient decision	36 (32.1)
Lack of efficacy	22 (19.6)
Tolerability/adverse event	9 (8.0)
Other	48 (42.9)

ALN‐EFF = buffered soluble alendronate 70 mg effervescent tablet.

^a^ Refers to previous antiosteoporotic medication.

### 3.2 Cumulative incidence of upper GI AEs

**Fig 2:**
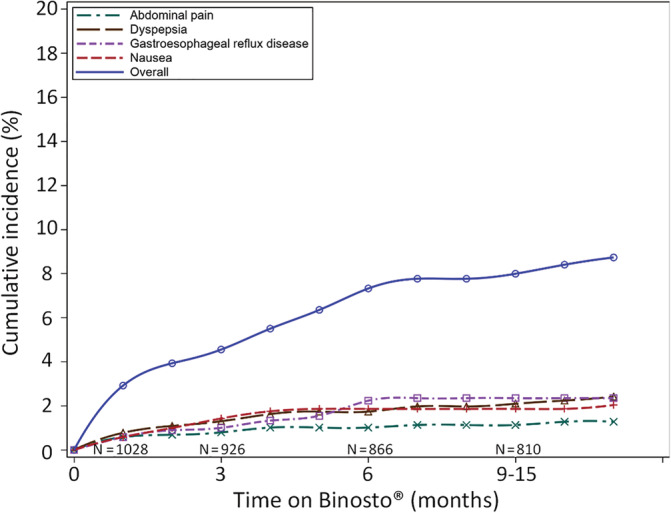
Kaplan–Meier plot showing the cumulative incidence of all upper gastrointestinal adverse events (AEs) related to buffered soluble alendronate 70 mg effervescent tablet (ALN‐EFF) over the follow‐up period. Specific AEs are also shown.

**Fig 3:**
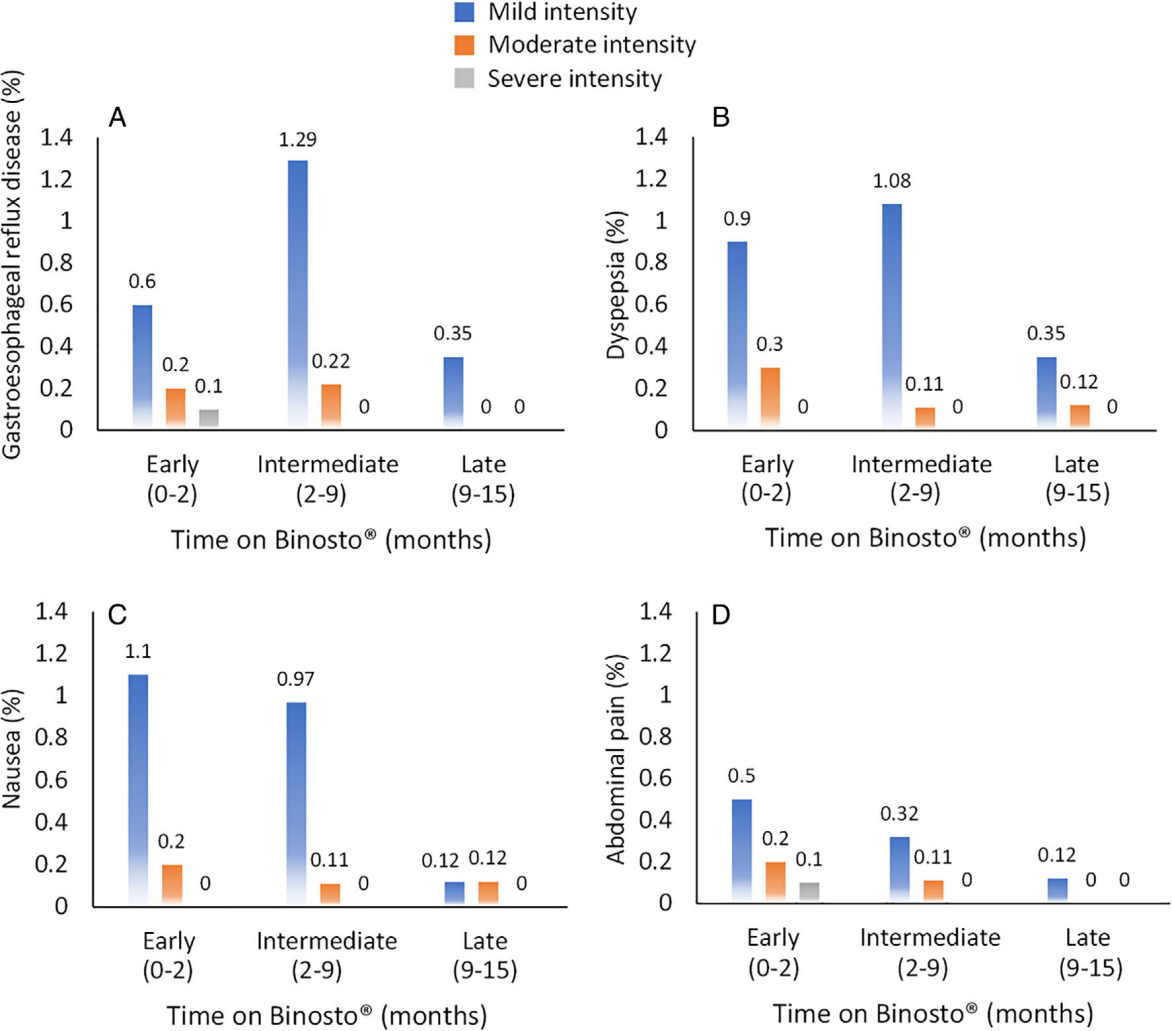
Incidence of upper gastrointestinal (GI) adverse events (AEs) over the follow‐up period. The % of upper GI AEs for gastroesophageal reflux disease (*A*), dyspepsia (*B*), nausea (*C*), and abdominal pain (*D*) are presented according to severity (mild, moderate, and severe) over the three visits (early, intermediate, and late follow‐up period).

The cumulative incidence of all upper GI AEs over the study period was 12.7% (95% CI 10.8–14.9%), whereas the cumulative incidence of upper GI AEs related to ALN‐EFF was 9.6% (CI 7.9–11.6%) (Fig. 2). Of these, 82 (8%; CI 6.4–9.8%) were of mild intensity, 15 (1.5%; CI 0.8–2.4%) of moderate intensity, and 2 (0.2%; CI 0.0–0.7%) of severe intensity (Table 3). The percentage of upper GI AEs related to ALN‐EFF reported at each visit decreased over the follow‐up period (Table 3). No serious upper GI AEs related to ALN‐EFF were reported over the entire study period. The most common upper GI AEs (≥1% of patients) related to ALN‐EFF were dyspepsia (2.7%; 28/1028), gastroesophageal reflux disease (2.4%; 25/1028), nausea (2.2%; 23/1028), and abdominal pain (1.3%; 13/1028). There were a total of 10 upper GI AEs that were considered by investigators to be related to ALN‐EFF with a cumulative incidence of <1%: gastritis (0.9%; 9/1028), all mild intensity; and duodenal ulcer (0.1%; 1/1028), of mild intensity on the background of a preexisting duodenal ulcer (“duodenal ulcer aggravated”). The incidence of specific upper GI AEs over the follow‐up period is shown in Fig. 3. The majority of events were mild at the early follow‐up and intermediate follow‐up, decreasing at the late follow‐up for all four upper GI AEs. There were no reports of any of the following AEs: esophagitis, esophageal ulcer, esophageal perforation, esophageal hemorrhage, esophageal stenosis, gastric ulcer, gastric perforation, gastric hemorrhage, gastric stenosis, duodenitis, duodenal perforation, small intestine hemorrhage, duodenal stenosis, or melaena.

**Table 3:** Cumulative Incidence of All Upper Gastrointestinal (GI) Adverse Events (AEs) and Individual Upper GI AEs by Follow‐up Time at the End of the Study

	Early follow‐up		Intermediate follow‐up		Late follow‐up		Overall	
	*n*	% (95% CI)	*n*	% (95% CI)	*n*	% (95% CI)	*n*	% (95% CI)
*n*	999	100	930	100	856	100	1028	100
All^a^	68	6.8 (5.3–8.6)	52	5.6 (4.2–7.3)	24	2.8 (1.8–4.1)	131	12.7 (10.8–14.9)
Related to ALN‐EFF	49	4.9 (3.7–6.4)	43	4.6 (3.4–6.2)	14	1.6 (0.9–2.7)	99	9.6 (7.9–11.6)
Mild intensity	38	3.8 (2.7–5.2)	37	4 (2.8–5.4)	12	1.4 (0.7–2.4)	82	8 (6.4–9.8)
Moderate intensity	8	0.8 (0.4–1.6)	6	0.7 (0.2–1.4)	2	0.2 (0.03–0.8)	15	1.5 (0.8–2.4)
Severe intensity	2	0.2 (0.02–0.7)	0	0 (0.0–0.0)	0	0 (0.0–0.0)	2	0.2 (0.02–0.7)

CI = confidence interval; ALN‐EFF = buffered soluble alendronate 70 mg effervescent tablet.

There were no reports during the study of any of the following: esophagitis, esophageal ulcer, esophageal perforation, esophageal hemorrhage, esophageal stenosis, gastric ulcer, gastric perforation, gastric hemorrhage, gastric stenosis, duodenitis, duodenal perforation, small intestine hemorrhage, duodenal stenosis, or melaena.

^a^ Patients with at least one gastrointestinal adverse event.

### 3.3 Factors associated with the incidence of upper GI AEs

A range of individual baseline characteristics were found to be associated with upper GI AEs using univariate analysis (Supplemental Table S2). After multivariate adjustment, only country (Italy had higher levels than Spain) with an odds ratio (OR) = 4.5 (CI 2.8–7.2%), history of dyspepsia with OR = 3.1 (CI 1.6–5.9%), history of gastritis with OR = 2.9 (CI 1.7–5.2%), and history of concomitant medication with OR = 1.8 (CI 1.1–3.1%) remained significant determinants of upper GI AEs. The high OR for Italy was attributed to one site that reported a high incidence of AEs, upper GI AEs, and other AEs.

### 3.4 Incidence of individual gastric AEs

The incidence of ALN‐EFF individual gastric AEs was low as shown in Table 4. The overall incidence rates (per 1000 patients/month) were 2.1 (CI 1.4–3.2) for nausea, 1.2 (CI 0.6–2.1) for abdominal pain, and 0.8 (CI 0.4–1.6) for gastritis (Table 4). The incidence rate also decreased over the three follow‐up periods for these three AEs. No events for gastric ulcer, gastric perforation, gastric hemorrhage, and gastric stenosis occurred.

**Table 4:** Incidence of Individual Gastrointestinal Adverse Events (AE) Related to ALN‐EFF Over the Follow‐up Period

AEs related to ALN‐EFF	Early follow‐up	Intermediate follow‐up	Late follow‐up	Overall
Nausea	7.8 (4.2–13.3)	2.1 (1–4.0)	0.2 (0.01–1.2)	2.1 (1.4–3.2)
Abdominal pain	1.8 (0.4–5.2)	1.2 (0.4–2.7)	0.2 (0.01–1.2)	1.2 (0.6–2.1)
Gastritis	4.8 (2.1–9.4)	0.9 (0.26–2.4)	0.2 (0.01–1.2)	0.8 (0.4–1.6)
Abdominal pain upper	0.6 (0.02–3.3)	0.9 (0.3–2.4)	0.4 (0.05–1.5)	0.6 (0.3–1.3)
Abdominal distension	3.6 (1.3–7.8)	0.0 (0.0–0.9)	0.0 (0.0–0.8)	0.56 (0.2–1.2)
Vomiting	0.6 (0.02–3.3)	0.0 (0.0–0.9)	0.2 (0.01–1.2)	0.2 (0.02–0.7)
*Helicobacter* gastritis	0.0 (0.0–2.2)	0.2 (0.01–1.3)	0.0 (0.0–0.8)	0.09 (0.0–0.3)
Gastric ulcer	0.0 (0.0–2.2)	0.0 (0.0–0.9)	0.0 (0.0–0.8)	0.0 (0.0–0.3)
Gastric perforation	0.0 (0.0–2.2)	0.0 (0.0–0.9)	0.0 (0.0–0.8)	0.0 (0.0–0.3)
Gastric hemorrhage	0.0 (0.0–2.2)	0.0 (0.0–0.9)	0.0 (0.0–0.8)	0.0 (0.0–0.3)
Gastric stenosis	0.0 (0.0–2.2)	0.0 (0.0–0.9)	0.0 (0.0–0.8)	0.0 (0.0–0.3)

ALN‐EFF = buffered soluble alendronate 70 mg effervescent tablet.

Incidence expressed as the rate per 1000 patients/month.

### 3.5 Medication errors

A total of 307 of 1028 patients (29.9%; CI 27.1–32.8%) made at least one medication error during the study with a total of 887 medication errors. The mean number of medication errors across all evaluable 1028 patients was 0.9 ± 7. From 1028 evaluable patients, 9.34% (CI 7.6–11.3%) made errors in the liquid used for dissolution (other liquid than plain water) and 0.97% (CI 0.5–1.8%) of patients in the volume of dissolution (<120 mL), 10.3% (CI 8.5–12.3%) made errors in the “waiting time before first meal” (intake of ALN‐EFF less than 30 minutes before the first meal of the day), 7.6% (CI 6.0–9.4%) made post‐administration drinking errors (not drinking at least 30 mL of plain water immediately after intake of the solution), 1.7% (CI 1.0–2.6%) made errors in the dosage (once weekly), 1.3% (CI 0.7–2.2%) made post‐administration behavior errors (stay upright less than 30′ after intake), 0.6% (6/1028; CI 0.2–1.3%) made errors in the complete dissolution of the preparation, and 0.1% (1/1028; CI 0.0–0.5%) used no liquid for dissolution. The majority of medication errors were associated with administration instructions applicable to any oral bisphosphonates in general (volume of liquid, 30 minutes before first meal, staying upright). Only seven medication errors were associated with an administration error specific to ALN‐EFF (six did not wait for the full dissolution of the preparation and one did not use liquid). Four medications errors were associated with AEs, three related to ALN‐EFF (two acid regurgitation and one gastritis of mild severity, non‐serious, with full recovery and continuation of ALN‐EFF). The third AE consisted of nausea of mild severity, non‐serious, related to ALN‐EFF, which resolved completely and reappeared upon administration of ALN‐EFF resulting in permanent discontinuation of ALN‐EFF. The number (and %) of patients making medication errors remained largely unchanged over the follow‐up period (Supplemental Table Table S3).

### 3.6 Discontinuation and compliance

**Fig 4:**
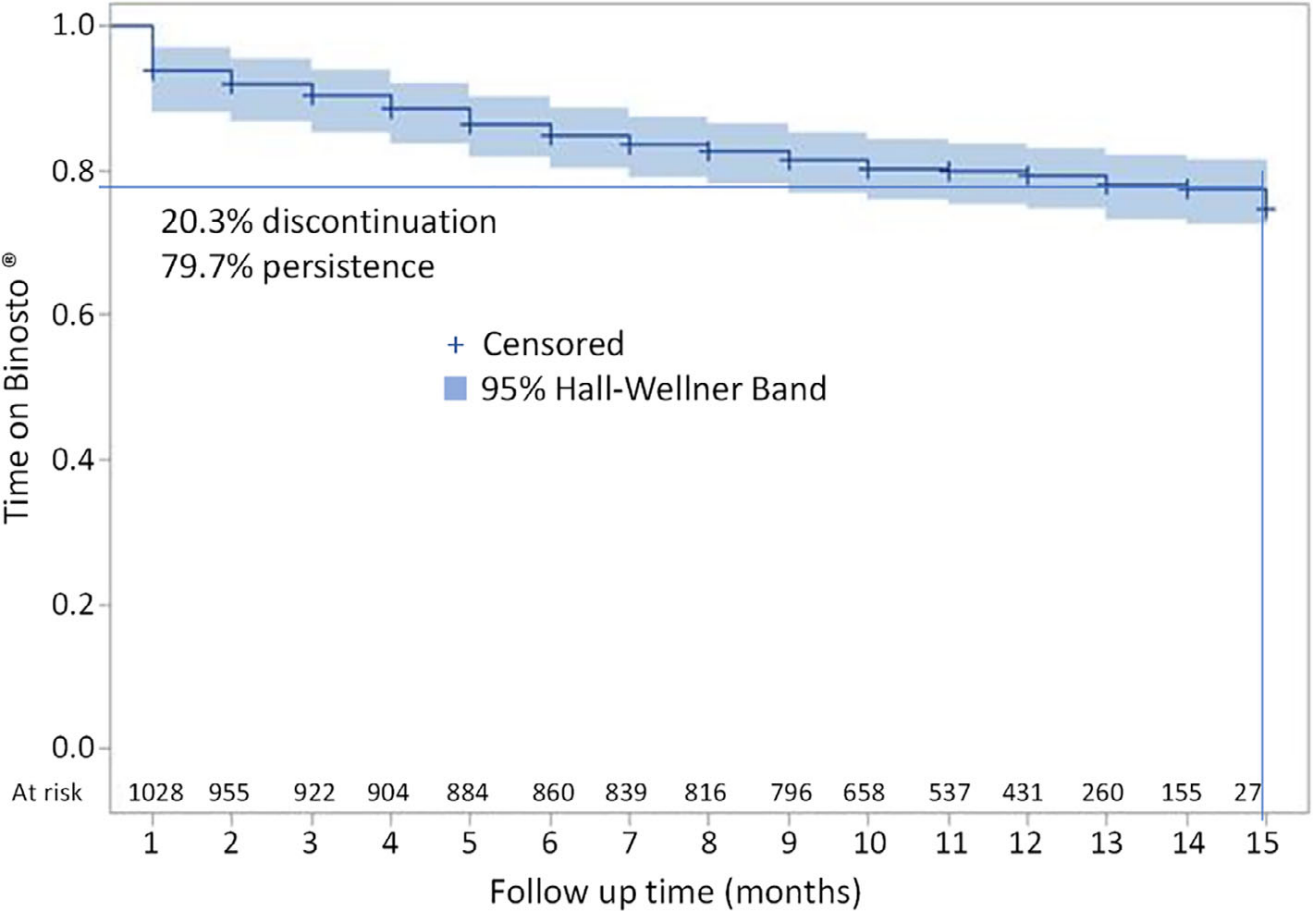
Kaplan–Meier plot showing the discontinuation rate of postmenopausal women treated with buffered soluble alendronate 70 mg effervescent tablet (ALN‐EFF) over the follow‐up period. Shaded area in light blue represents lower and upper 95% confidence interval. Number of patients at risk at each time point are shown on the *x* axis.

The mean time on ALN‐EFF was 12.8 months (CI 12.6–12.9). Of 1028 evaluable patients, 209 (20.3%) permanently discontinued ALN‐EFF over the follow‐up period. Discontinuation decreased steadily over time; 9.6% at 3 months, 6.0% at 6 months, 4.1% at 9 months, 2.4% at 12 months, and 2.3% at 15 months (Fig. 4). The most common reasons were AEs related to ALN‐EFF (46.9%; 98/209) and patient decision (42.6%; 89/209). Discontinuation for other causes constituted 24.9% (52/209) and 0.5% (1/209) were due to lack of efficacy. Among the 52 patients who discontinued for other causes, 14 (26.9%) discontinued due to dental care, 11 (21.2%) due to shortage of drug supplies, 8 (15.4%) for changes in treatment, 7 (13.5%) due to medical advice, 7 (13.5%) for other concomitant conditions, 4 (7.7%) due to patient condition, and 1 (1.9%) for other treatment issues. Multivariate logistic regression found age to be associated with discontinuation of ALN‐EFF due to AEs (OR = 0.96; CI 0.94–1.00).

The mean overall compliance using the Morisky‐Green questionnaire was 92.8 ± 8.6 and the mean overall compliance based on the number of tablets missed was 94.8 ± 8.1. Patient compliance did not change over the follow‐up period (Supplemental Table Table S4).

Multivariate logistic regression found that the only variables to be associated with treatment compliance with ALN‐EFF were country (improved compliance in Italy versus Spain; OR = 1.66; CI 1.2–2.4) and less compliance in patients with a history of GI symptoms (OR = 0.67; CI 0.5–1.0). Patient satisfaction remained high (approximately 90%) in terms of how easy it was to take ALN‐EFF and how much easier it was to take compared with other medications (Supplemental Table Table S5).

### 3.7 Reasons for discontinuation over the follow‐up period

The most frequent reason for discontinuation, the presence of AEs, decreased over the follow‐up period, 38/53 patients (71.7%) at the early follow‐up, 45/71 patients (52.9%) at the intermediate follow‐up, and 15/98 patients (21.1%) at the late follow‐up. Only one patient discontinued due to lack of efficacy (at the late follow‐up visit) (Supplemental Table S6).

## 4 Discussion

This postauthorization safety study (PASS) evaluated the incidence of upper GI AEs and medication errors of a novel buffered soluble alendronate 70 mg effervescent tablet formulation (ALN‐EFF) in PMW over 1 year in Italy and Spain. Results from this study provide evidence for the safe real‐world use of ALN‐EFF in bisphosphonate‐naïve PMW with osteoporosis.

A total of 23% of patients experienced at least one AE during the study, while only 12.5% of patients reported AEs that were deemed to be related to ALN‐EFF. The incidence of AEs was similar to another real‐life experience with oral bisphosphonates.^(^ ^27^ ^)^ In addition, only a few AEs were serious, and no serious AE were related to ALN‐EFF. The vast majority (96%) of AEs related to ALN‐EFF were of mild or moderate intensity, and a high level of satisfaction with taking ALN‐EFF was found at both early and late follow‐up.

The cumulative incidence of all upper GI AEs during this 1‐year study was 12.7%, with the majority of these (9.6%) considered to be related to ALN‐EFF. Upper GI AEs related to ALN‐EFF decreased with time and were almost all of mild‐to‐moderate intensity with only two judged as severe, both of brief duration with full patient recovery.

These results cannot directly be compared with other studies because of the lack of a control group and potential differences in the patient population (baseline characteristics) from other studies. However, the incidence of upper GI AEs in this “real‐world” study were found to be lower than that observed in alendronate‐treated groups from randomized controlled trials with a duration of 12 months.

In the FIT study (5 mg ALN daily in the first 2 years, then 10 mg/d), the incidence in the first 12 months was about 30%.^(^ ^13^ ^)^ In a second 12‐month trial using a dose of 10 mg/d, the incidence was 21.3% over 12 months.^(^ ^14^ ^)^ Two further trials that continued 70 mg once weekly for at least 1 year (ie, similar to ALN‐EFF) with incidence of upper GI AEs of 23.5%^(^ ^16^ ^)^ in one and 22.5%^(^ ^15^ ^)^ in the other. A 3‐month safety study of 70 mg once weekly reported an incidence of 11% over 3 months.^(^ ^28^ ^)^ This evidence is consistent with the conclusion that the ALN‐EFF dosing regimen may result in lower incidence of upper GI events over 1 year than a pill at the same dose.

The incidence of more concerning upper GI events (ie, ulcer, perforation, hemorrhage, stenosis, etc.) were not reported. In this regard, the physician would have certainly prescribed an esophagogastroduodenoscopy in cases where the patients would have presented severe clinical symptoms.

At least one medication error occurred in 29.9% of patients and only seven medication errors were associated with an administration error specific to the new ALN‐EFF formulation, while the vast majority of administration errors in medication observed were common to the administration procedure for any oral bisphosphonate.^(^ ^29^ ^)^ In this regard, Ettinger and colleagues reported in an analysis of 812 women that around 60% did not comply with at least one of the alendronate dosing instructions, mostly waiting 30 minutes before food and using liquid other than water. These two dosing instruction errors were observed in two‐thirds of patients reporting medication errors.^(^ ^29^ ^)^ However, the results of our study show that the use of the ALN‐EFF‐specific alendronate formulation is not associated with an increased risk of medication errors compared with standard oral alendronate formulations. It is recognized that instructions for proper administration of bisphosphonates are rather complex and require adequate education from health care professionals to patients, particularly in elderly people.^(^ ^30^ ^)^

Only 4 patients reported AEs associated with medication errors, which were of mild severity, non‐serious, and all recovered. Only in one case did this lead to discontinuation of ALN‐EFF. Improper administration (correct amount of water or staying in upright position for 30′) of alendronate has been associated with esophageal mucosal injury, especially in post marketing reports.^(^ ^10^, ^13^ ^)^ This was not observed in this study of more than 1000 patients.

The incidence of ALN‐EFF individual gastric AEs was low, ranging from 0% to 2.1% per 1000 patients/month. Large prescription‐event monitoring studies have been carried out in England for alendronate in PMW with osteoporosis.^(^ ^31^, ^32^ ^)^ In the first study involving 1523 patients, 20 (1.3%) experienced an esophageal event that was possibly related to alendronate.^(^ ^31^ ^)^ In the second study, involving 11,916 patients, the incidence density for the overall treatment period (IDA) for dyspeptic conditions was 10.1 per 1000 patients/month, nausea and vomiting 4.8 per 1000 patients/months, and abdominal pain 3.8 per 1000 patients/month.^(^ ^32^ ^)^ Based on these studies, a report commissioned by the UK National Health Service concluded that conventional alendronate appeared to be associated with GI complaints but with some degree of uncertainty due to the lack of a placebo arm. Despite the absence of a direct comparison between these ALN‐EFF study results, the IDA for nausea and vomiting and abdominal pain appeared to be lower for ALN‐EFF.

It is recognized that approximately three‐quarters of women who initiate bisphosphonates are nonadherent to treatment and almost 50% discontinue therapy within the first year.^(^ ^8^, ^9^ ^)^ For ALN‐EFF, a lower discontinuation was observed in this study, with approximately one‐fifth (20.3%) of patients permanently discontinuing treatment. The decline in discontinuation in the first month was larger followed by a time‐dependent steady decline thereafter. This supports the observation that people who have problems with tolerability or experience early side effects tend to stop treatment early. The interpretation of these results is in line with previous publications where patients who interrupt therapy with bisphosphonates do so because of drug‐induced AEs. Undoubtedly, among these AEs, those affecting the upper GI system are by far the most frequent.^(^ ^9^ ^)^ Nonetheless, the comparably lower discontinuation rate due to tolerability and AEs may be the best indicator of the real‐world experience of ALN‐EFF in this study.

After multivariable adjustment, only history of dyspepsia, history of gastritis, and history of concomitant medication remained as significant determinants of upper GI events. These results are consistent with other studies,^(^ ^13^ ^)^ where previous upper GI tract disease was identified as an important independent risk factor for upper GI tract events. Though the perception is that alendronate frequently causes GI tract intolerance, it is likely that upper GI complaints are common among elderly women, as observed in the FIT study,^(13^ ^)^ regardless of alendronate treatment or placebo.

In addition, differences in results between Spain and Italy may indicate cultural differences and health care system can affect the reporting of AEs. Nonetheless, the results of this analysis should be applicable to a wide range of PMW with osteoporosis who are naïve to bisphosphonates according to the SmPC.

To date, only one study has evaluated the effect of ALN‐EFF compared with standard ALN tablets on bone mineral density (BMD) and bone‐alkaline phosphatase (b‐ALP).^(^ ^33^ ^)^ A preliminary study by Giusti and colleagues involving 42 PMW with BMD *T*‐score <−2.5 (or between −2 and −2.5 and at least one vertebral fracture) were treated with ALN‐EX for 12 months and compared with 54 PMW on alendronate standard tablet randomly selected from a historical cohort. After 12 months of treatment, the two groups showed a similar increase in femoral neck and total hip BMD. The absolute decrease (mean U/L ± SD) of b‐ALP was also comparable between the ALN‐EX group (−6.0 ± 2.8 U/L) and the standard alendronate group (−6.9 ± 4.4 U/L). These preliminary results demonstrated that ALN‐EFF is as effective as traditional alendronate tablet on surrogate anti‐fracture efficacy outcomes.

### 4.1 Study strengths

The strengths of the present study are that this was a real‐world assessment of upper GI AEs in a wide variety of clinical settings in a large number (*N* = 1028) of PMW in two EU countries.

Although participating physicians may not be representative of all physicians who were prescribing ALN‐EFF, the wide spectrum of sites (primary and secondary care centers) and consecutive recruitment of eligible patients minimized any systematic selection bias and may permit generalizability of the results to populations in Spain and Italy.

Medication errors were assessed in routine clinical practice and reasons for discontinuation of ALN‐EFF were systematically assessed. At baseline, 31% of enrolled patients reported a medical history of GI tract conditions that did not exclude study participation, and 85% of these patients presented with a history of upper GI symptoms. The limited exclusion criteria may be a better reflection of real‐world experience with ALN‐EFF compared with previous clinical studies with oral alendronates with more restrictive exclusion criteria.^(^ ^34^ ^)^

This observational (PASS) design separated the prescription from the decision to include a patient in the study, followed current clinical practice, and allows for the inclusion of a wide range of patients who used ALN‐EFF.

### 4.2 Study limitations

The main limitation of the present study was the lack of a control group with weekly 70 mg alendronate in a similar real‐world setting to compare the incidence of upper GI AEs, persistence, and discontinuation with ALN‐EFF.

Events observed were symptomatic AEs, with any further investigation determined as necessary during routine clinical practice.

Loss to follow‐up was different between Italy and Spain according to different clinical practice in the two countries and due to differences in the wording of the country‐specific case report forms.

Data collection on the intensity of AEs was requested after recruitment and data collection had already started, so data were not available for all patients enrolled.

Assessment of adherence was limited in patients recorded as having temporary discontinuations of treatment.

The small number of patients and events in some patient subgroups meant that the assessment of many secondary and exploratory objectives was limited. Therefore, results derived from some of these subgroup analyses need to be interpreted with caution.

## Conclusion

Based on the results of this study, it is suggested that ALN‐EFF may represent an alendronate treatment for patients with postmenopausal osteoporosis that is associated with preferable pharmacologic properties translating into clinical benefit for patients. Many of the upper GI AEs of interest (primary objective) were observed to occur at a lower incidence compared with the reported frequency from randomized controlled trials and post‐marketing data in the SmPC of the reference product (alendronate tablets).^(^ ^35^ ^)^ Furthermore, ALN‐EFF was not associated with a detectable increase in the risk of serious upper GI tract AEs.

This PASS was initiated in order to generate real‐world data on this new formulation of alendronate, a buffered, oral solution. The results provide evidence that ALN‐EFF does not lead to a higher incidence of upper GI/esophageal AEs compared with standard alendronate tablets and despite its somewhat different mode of preparation/administration versus standard alendronate tablets does not lead to an increased incidence of administration errors.

Further studies beyond 12 months to determine whether persistence can be maintained and whether increased persistence translates into improved long‐term efficacy (eg, increase in bone mineral disease, incidence of secondary fractures, etc.) are warranted.

Finally, having a drug with negligible side effects allows long‐term adherence. This is of utmost importance in a pandemic period in which patients are reluctant to go to hospitals or to outpatient clinics.

## Disclosures

GA reports personal fees for Amgen and Theramex. DMB reports personal fees from Merck, Amgen, Asahi‐Kasei, Eli Lilly, and Effx during the conduct of the study and personal fees from University of Pittsburgh outside the submitted work. JBR has received grants or consulting fees from Amgen SA, Laboratorio Stada SL, Gedeon‐Rhicter Ibérica, Lilly España, SA, Pfizer, Gebro Pharma, and UCB Pharma SA. SM served as speaker for Abiogen, Amgen, Bruno Farmaceutici, Diasorin, Eli Lilly, Shire, Sandoz, and Takeda. He has also served on advisory boards for Abiogen, Kyowa Kirin, Pfizer, and UCB. APV has received research fees from Laboratories Lacer. GLM and FBM have no conflicts of interest to declare. NQ is the owner of OXON Epidemiology, which was funded to conduct and analyze this study.

## Author contributions

**Salvatore Minisola:** Investigation; methodology; project administration; supervision; visualization; writing‐original draft; writing‐review & editing. **Antonio Vargas:** Investigation; methodology; project administration; supervision; visualization; writing‐original draft; writing‐review & editing. **Giulia Letizia Mauro:** Investigation; methodology; project administration; supervision; visualization; writing‐original draft; writing‐review & editing. **Fernando Bonet Madurga:** Investigation; methodology; project administration; supervision; visualization; writing‐original draft; writing‐review & editing. **Giovanni Adami:** Investigation; methodology; project administration; supervision; visualization; writing‐original draft; writing‐review & editing. **Dennis Black:** Conceptualization; investigation; methodology; project administration; supervision; visualization; writing‐original draft; writing‐review & editing. **Nawab Qizilbash:** Data curation; formal analysis; methodology; software; validation; writing‐original draft; writing‐review & editing. **Josep Blanch‐Rubió:** Investigation; methodology; project administration; supervision; visualization; writing‐original draft; writing‐review & editing.

5

### Peer review

The peer review history for this article is available at https://publons.com/publon/10.1002/jbm4.10510.

## Supporting information


**Supplemental Table S1.** Questionnaire to Capture Potential Errors in the Method of Administration of Binosto

**Supplemental Table S2**. Association of Baseline Characteristics With Upper Gastrointestinal Events During the Follow‐up Period

**Supplemental Table S3.** Description of Responses to the Questionnaire for the Identification of Possible Medication Errors at Early, Intermediate, and Late Follow‐up

**Supplemental Table S4.** Compliance Over the Follow‐up Period According to the Morisky‐Green Questionnaire and Number of Tablets Missed

**Supplemental Table S5.** Patient Satisfaction During the Follow‐up Period

**Supplemental Table S6.** Discontinuation and Reasons for Discontinuation, by Follow‐up Time Window and at the End of the Study
