# Supplementary material for: A Prospective Open‐Label Observational Study of a Buffered Soluble 70 mg Alendronate Effervescent Tablet on Upper Gastrointestinal Safety and Medication Errors: The GastroPASS Study
Source: JBMR Plus. 2021 May 17;5(7):e10510. doi: 10.1002/jbm4.10510 (PMC8260812; doi:10.1002/jbm4.10510)
Supplement: Supplementary file 1 — Supplemental Table S1. Questionnaire to Capture Potential Errors in the Method of Administration of Binosto [file JBM4-5-e10510-s002.docx]

**Supplementary Material S1.** Questionnaire to capture potential errors in the method of administration of Binosto®

**Q1) Have you dissolved the Binosto® tablet in at least half a glass (120 ml) of plain water**

**before taking it?**

Yes (go to Q3)

No (go to Q2)

**Q2) Which liquid did you use for dissolution?**

Plain water, but less than half a glass (less than 120 ml) was used.

Other liquid >  mineral water juice milk coffee other (specify)…………………

No liquid was used and Binosto® tablet was swallowed or chewed (Go to Q 7)

**Q3) Did you allow the Binosto® tablet to completely dissolve in the liquid?**

Yes

No

**Q4) Did you drink at least one sixth of a glass (30 ml) of plain water immediately after**

**intake of the solution?**

Yes

No

**Q5) Have you taken Binosto® at least 30 minutes before the first meal of the day?**

Yes

No

**Q6) Have you stayed upright for at least 30 minutes after taking Binosto®?**

Yes

No

______________________________________________________________________________

If any of the above Q is answered NO ensure that Q7 and Q8 are also answered

______________________________________________________________________________

**Q7) Has/have error/s in methods of administration resulted in adverse events?**

Yes (Go to Q8)

No

**Q8) Specify what adverse event occurred as result of medication error/s and ensure that the adverse events is linked to a medication error**
