# Supplementary material for: A Prospective Open‐Label Observational Study of a Buffered Soluble 70 mg Alendronate Effervescent Tablet on Upper Gastrointestinal Safety and Medication Errors: The GastroPASS Study
Source: JBMR Plus. 2021 May 17;5(7):e10510. doi: 10.1002/jbm4.10510 (PMC8260812; doi:10.1002/jbm4.10510)
Supplement: Supplementary file 2 — Supplemental Table S2. Association of Baseline Characteristics With Upper Gastrointestinal Events During the Follow‐up Period [file JBM4-5-e10510-s004.docx]

Supplementary Material Table S2. Association of baseline characteristics with upper gastrointestinal events during the follow-up period

|  |  | Any upper gastrointestinal AE at any time during follow-up | | | | | | | |
| --- | --- | --- | --- | --- | --- | --- | --- | --- | --- |
|  |  | Yes | | No |  |  |  |  |  |
| Categorical variables | Valid n (%) | n | % | n | % | p-value |  |  |  |
| Country |  |  |  |  |  |  |  |  |  |
| - Italy | 382 (37.2) | 85 | 8.3 | 297 | 28.9 | <0.001 |  |  |  |
| - Spain | 646 (62.8) | 46 | 4.5 | 600 | 58.4 |  |  |  |  |
| Education level |  |  |  |  |  |  |  |  |  |
| - No schooling completed | 51 (5) | 4 | 0.4 | 47 | 4.6 | 0.068 |  |  |  |
| - Primary School | 459 (44.7) | 51 | 5 | 408 | 39.8 |  |  |  |  |
| - Secondary school | 353 (34.4) | 59 | 5.8 | 294 | 28.7 |  |  |  |  |
| - Bachelor´s degree | 149 (14.5) | 17 | 1.7 | 132 | 12.9 |  |  |  |  |
| - Other professional qualification | 14 (1.4) | 0 | 0.0 | 14 | 1.4 |  |  |  |  |
| Employment status* |  |  |  |  |  |  |  |  |  |
| - Worker | 244 (23.8) | 43 | 4.2 | 201 | 19.6 | 0.018 |  |  |  |
| - Retired | 506 (49.4) | 52 | 5.1 | 454 | 44.3 |  |  |  |  |
| - Unemployed | 274 (26.8) | 36 | 3.5 | 238 | 23.2 |  |  |  |  |
| Smoking habits |  |  |  |  |  |  |  |  |  |
| - Ex-smoker | 139 (13.6) | 18 | 1.8 | 121 | 11.8 | 0.959 |  |  |  |
| - Never smoked | 759 (74.2) | 98 | 9.6 | 661 | 64.6 |  |  |  |  |
| - Current smoker | 125 (12.2) | 15 | 1.5 | 110 | 10.8 |  |  |  |  |
| History of diabetes (1 or 2 type) |  |  |  |  |  |  |  |  |  |
| -Yes | 88 (8.6) | 5 | 0.49 | 83 | 8.1 | 0.043 |  |  |  |
| -No | 940 (91.4) | 126 | 12.26 | 814 | 79.2 |  |  |  |  |
| History of thyroid / parathyroid disorder |  |  |  |  |  |  |  |  |  |
| -Yes | 154 (15) | 28 | 2.72 | 126 | 12.3 | 0.035 |  |  |  |
| -No | 874 (85) | 103 | 10.02 | 771 | 75 |  |  |  |  |
| History of inflammatory bowel disease |  |  |  |  |  |  |  |  |  |
| -Yes | 16 (1.6) | 5 | 0.5 | 11 | 1.1 | 0.042 |  |  |  |
| -No | 1012 (98.4) | 126 | 12.3 | 886 | 86.2 |  |  |  |  |
| History of dyspepsia |  |  |  |  |  |  |  |  |  |
| -Yes | 108 (10.5) | 22 | 2.1 | 86 | 8.4 | 0.021 |  |  |  |
| -No | 920 (89.5) | 109 | 10.6 | 811 | 78.9 |  |  |  |  |
| History of acid regurgitation |  |  |  |  |  |  |  |  |  |
| -Yes | 98 (9.5) | 24 | 2.3 | 74 | 7.2 | <0.001 |  |  |  |
| -No | 930 (90.5) | 107 | 10.4 | 823 | 80.1 |  |  |  |  |
| History of gastritis |  |  |  |  |  |  |  |  |  |
| -Yes | 103 (10) | 32 | 3.1 | 71 | 6.9 | <0.001 |  |  |  |
| -No | 925 (90) | 99 | 9.6 | 826 | 80.4 |  |  |  |  |
| History of gastric ulcers |  |  |  |  |  |  |  |  |  |
| -Yes | 12 (1.2) | 5 | 0.5 | 7 | 0.7 | 0.012 |  |  |  |
| -No | 1016 (98.8) | 126 | 12.3 | 890 | 86.6 |  |  |  |  |
| History of osteoporosis treatment |  |  |  |  |  |  |  |  |  |
| -Yes | 111 (10.8) | 10 | 1 | 101 | 9.8 | 0.29 |  |  |  |
| -No | 917 (89.2) | 121 | 11.8 | 796 | 77.4 |  |  |  |  |
| History of osteoporosis supplement |  |  |  |  |  |  |  |  |  |
| -Yes | 625 (60.8) | 97 | 9.4 | 528 | 51.4 | 0.001 |  |  |  |
| -No | 403 (39.2) | 34 | 3.3 | 369 | 35.9 |  |  |  |  |
| History of a concomitant medication ^#^ |  |  |  |  |  |  |  |  |  |
| -Yes | 724 (76) | 96 | 10.1 | 628 | 65.9 | 0.5 |  |  |  |
| -No | 229 (24.0) | 26 | 2.7 | 203 | 21.3 |  |  |  |  |
| Continuous variables |  |  |  |  |  |  |  |  |  |
|  | Valid n (%) | n (Mean) | Std. Error | n (Mean) | Std. Error | p | t/Z statistic |  |  |
| Age (years) | 1028 (100) | 131 (64.6) | 8.9 | 897 (67.7) | 9.4 | 0.001 | 3.5 |  |  |
| Time since menopause (years) | 875 (100) | 123 (15.1) | 9.7 | 752 (17.7) | 9.9 | 0.007 | -2.7 |  |  |

| * For this variable the categories student, self-employed and house-wife were grouped with categories worker and unemployed respectively | | | | |
| --- | --- | --- | --- | --- |
| ^#^ For this variable the category “unknown” was excluded from the analysis |  |  |  |  |
