# Supplementary material for: A Prospective Open‐Label Observational Study of a Buffered Soluble 70 mg Alendronate Effervescent Tablet on Upper Gastrointestinal Safety and Medication Errors: The GastroPASS Study
Source: JBMR Plus. 2021 May 17;5(7):e10510. doi: 10.1002/jbm4.10510 (PMC8260812; doi:10.1002/jbm4.10510)
Supplement: Supplementary file 3 — Supplemental Table S3. Description of Responses to the Questionnaire for the Identification of Possible Medication Errors at Early, Intermediate, and Late Follow‐up [file JBM4-5-e10510-s003.docx]

Supplementary Material S3. Description of responses to the questionnaire for the identification of possible medication errors at early, intermediate and late follow-up

|  | ALN-EFF | | | | | | | | |
| --- | --- | --- | --- | --- | --- | --- | --- | --- | --- |
|  | Early follow-up | | Intermediate follow-up | | | Late follow-up | | |  |
|  | N | % | | N | % | | N | % | |
| N* | 943 | 100 | | 842 | 100 | | 785 | 100 | |
| ALN-EFF tablet dissolved in at least half a glass (120ml) of plain water? |  |  | |  |  | |  |  | |
| - Yes | 854 | 90.6 | | 771 | 91.6 | | 726 | 92.5 | |
| - No | 89 | 9.4 | | 71 | 8.4 | | 59 | 7.5 | |
| - Missing | 0 |  | | 0 |  | | 0 |  | |
| Liquid used for dissolution |  |  | |  |  | |  |  | |
| - Plain water | 7 | 7.9 | | 2 | 2.8 | | 1 | 1.7 | |
| - Other liquid | 81 | 91.0 | | 69 | 97.2 | | 58 | 98.3 | |
| + Mineral water | 76 | 96.2 | | 69 | 100 | | 58 | 100 | |
| + Juice | 1 | 1.3 | | 0 | 0.0 | | 0 | 0.0 | |
| + Milk | 0 | 0.0 | | 0 | 0.0 | | 0 | 0.0 | |
| + Coffee | 0 | 0.0 | | 0 | 0.0 | | 0 | 0.0 | |
| + Other | 2 | 2.5 | | 0 | 0.0 | | 0 | 0.0 | |
| + Missing | 2 |  | | 0 |  | | 0 |  | |
| - No liquid (swallowed or chewed) | 1 | 1.1 | | 0 | 0.0 | | 0 | 0.0 | |
| - Missing | 0 |  | | 0 |  | | 0 |  | |
| Tablet dissolved completely |  |  | |  |  | |  |  | |
| - Yes | 852 | 99.9 | | 771 | 100 | | 726 | 100 | |
| - No | 1 | 0.1 | | 0 | 0.0 | | 0 | 0.0 | |
| - Missing | 1 |  | | 0 |  | | 0 |  | |
| Drink at least 30mL of plain water after intake |  |  | |  |  | |  |  | |
| - Yes | 897 | 95.2 | | 810 | 96.3 | | 761 | 96.9 | |
| - No | 45 | 4.8 | | 31 | 3.7 | | 24 | 3.1 | |
| - Missing | 1 |  | | 1 |  | | 0 |  | |
| ALN-EFF taken at least 30 min before first meal of the day |  |  | |  |  | |  |  | |
| - Yes | 851 | 90.3 | | 751 | 89.2 | | 705 | 89.8 | |
| - No | 91 | 9.7 | | 91 | 10.8 | | 80 | 10.2 | |
| - Missing | 1 |  | | 0 |  | | 0 |  | |
| Stay upright at least 30min after intake |  |  | |  |  | |  |  | |
| - Yes | 938 | 99.6 | | 840 | 99.8 | | 784 | 99.9 | |
| - No | 4 | 0.4 | | 2 | 0.2 | | 1 | 0.1 | |
| - Missing | 1 |  | | 0 |  | | 0 |  | |
| Taken ALN-EFF only once a week |  |  | |  |  | |  |  | |
| - Yes | 939 | 99.6 | | 840 | 99.8 | | 784 | 99.9 | |
| - No | 4 | 0.4 | | 2 | 0.2 | | 1 | 0.1 | |
| - Missing | 0 |  | | 0 |  | | 0 |  | |
| Medication error/s resulted in AEs |  |  | |  |  | |  |  | |
| - Yes | 1 | 0.5 | | 0 | 0.0 | | 0 | 0.0 | |
| - No | 216 | 99.5 | | 194 | 100 | | 165 | 100 | |
| - Missing | 0 |  | | 0 |  | | 0 |  | |

* N stands for 'Patients on ALN-EFF'

ALN-EFF: buffered soluble alendronate 70 mg effervescent tablet
