# Supplementary material for: A Prospective Open‐Label Observational Study of a Buffered Soluble 70 mg Alendronate Effervescent Tablet on Upper Gastrointestinal Safety and Medication Errors: The GastroPASS Study
Source: JBMR Plus. 2021 May 17;5(7):e10510. doi: 10.1002/jbm4.10510 (PMC8260812; doi:10.1002/jbm4.10510)
Supplement: Supplementary file 4 — Supplemental Table S4. Compliance Over the Follow‐up Period According to the Morisky‐Green Questionnaire and Number of Tablets Missed [file JBM4-5-e10510-s005.docx]

Supplementary Material S4. Compliance over the follow-up period according to the Morisky-Green questionnaire and number of tablets missed.

|  | Early follow-up | | Intermediate follow-up | | Late follow-up | | Overall | |
| --- | --- | --- | --- | --- | --- | --- | --- | --- |
|  |  |  |  |  |  |  |  |  |
|  | N | % | N | % | N | % | N | % |
| N | 999 | 100 | 930 | 100 | 856 | 100 | 1028 | 100 |
| Patients on ALN-EFF | 943 | 94.4 | 842 | 90.5 | 785 | 91.7 | 815 | 79.3 |
| Compliance with ALN-EFF |  |  |  |  |  |  |  |  |
| Compliance based on Morisky-Green scale | 942 | 99.9 | 824 | 97.9 | 768 | 91.2 | 798 | 97.9 |
| - High adherence | 859 | 91.2 | 724 | 87.9 | 676 | 88.0 | 702 | 88 |
| - Medium adherence | 80 | 8.5 | 98 | 11.9 | 92 | 12 | 60 | 7.5 |
| - Low adherence | 3 | 0.3 | 2 | 0.2 | 0 | 0.0 | 36 | 4.5 |
| - Missing | 1 |  | 18 |  | 17 |  | 0 |  |
|  |  |  |  |  |  |  |  |  |
| Overall compliance (n)* |  |  |  |  |  |  | 798 | |
| Mean (standard deviation) |  |  |  |  |  |  | 92.80 (18.6) | |
|  |  |  |  |  |  |  |  |  |
| Compliance based on the number of tablets missed | 943 | 100 | 824 | 97.9 | 768 | 91.2 | 798 | 97.9 |
| - High adherence | 901 | 95.6 | 814 | 98.8 | 760 | 99 | 755 | 94.6 |
| - Medium adherence | 29 | 3.1 | 9 | 1.09 | 6 | 0. 8 | 6 | 0.8 |
| - Low adherence | 13 | 1.4 | 1 | 0.1 | 2 | 0.3 | 37 | 4.6 |
| - Missing | 0 |  | 18 |  | 17 |  | 0 |  |
|  |  |  |  |  |  |  |  |  |
| Overall compliance (n)* |  |  |  |  |  |  | 798 | |
| Mean (standard deviation) |  |  |  |  |  |  | 94.8 (18.1) | |

ALN-EFF: buffered soluble alendronate 70 mg effervescent tablet
