# Supplementary material for: A Prospective Open‐Label Observational Study of a Buffered Soluble 70 mg Alendronate Effervescent Tablet on Upper Gastrointestinal Safety and Medication Errors: The GastroPASS Study
Source: JBMR Plus. 2021 May 17;5(7):e10510. doi: 10.1002/jbm4.10510 (PMC8260812; doi:10.1002/jbm4.10510)
Supplement: Supplementary file 5 — Supplemental Table S5. Patient Satisfaction During the Follow‐up Period [file JBM4-5-e10510-s001.docx]

Supplementary Material S5. Patient satisfaction during the follow-up period

|  | Early follow-up | | Intermediate follow-up | | Late follow-up | |
| --- | --- | --- | --- | --- | --- | --- |
|  | N | % | N | % | N | % |
| N* | 943 | 100 | 842 | 100 | 785 | 100 |
| Do you find ALN-EFF easy to take? |  |  |  |  |  |  |
| - Yes | 858 | 91.7 | 747 | 88.9 | 694 | 88.6 |
| + Reasons for easiness (if freq > 10%) |  |  |  |  |  |  |
| + Dissolves in water | 271 | 31.7 | 213 | 28.6 | 201 | 29.1 |
| + Not specified | 143 | 16.7 | 128 | 17.2 | 113 | 16.4 |
| + Easy to take | 115 | 13.4 | 132 | 17.7 | 95 | 13.8 |
| - No | 76 | 8.1 | 93 | 11.1 | 89 | 11.4 |
| + Reasons for non-easiness (if freq > 10%) |  |  |  |  |  |  |
| + Prefers tablet | 21 | 27.6 | 42 | 45.2 | 64 | 71.9 |
| + The wait | 14 | 18.4 | 0 | 0.0 | 0 | 0.0 |
| + Fasting | 8 | 10.5 | 0 | 0.0 | 0 | 0.00 |
| + Staying upright | 8 | 10.5 | 0 | 0.0 | 0 | 0.0 |
| + Inconvenient to take | 0 | 0.0 | 10 | 10.8 | 0 | 0.0 |
| - Missing | 9 |  | 2 |  | 2 |  |
| Easier / more difficult than your other medications |  |  |  |  |  |  |
| - Easier | 758 | 93.9 | 696 | 93.9 | 639 | 97.4 |
| - More difficult | 49 | 6.1 | 45 | 6.1 | 17 | 2.6 |
| + Reasons for difficulties (if freq > 10%) |  |  |  |  |  |  |
| + Staying upright | 7 | 19.4 | 6 | 17.1 | 2 | 15.4 |
| + Fasting | 6 | 16. 7 | 0 | 0.0 | 0 | 0.0 |
| + The wait / Fasting | 5 | 13. 9 | 0 | 0.0 | 2 | 15.4 |
| + Prefers tablet | 4 | 11.1 | 6 | 17.1 | 0 | 0.0 |
| + The wait | 4 | 11.1 | 0 | 0.0 | 0 | 0.0 |
| + Inconvenient to take | 0 | 0.0 | 7 | 20.0 | 2 | 15.4 |
| + Effervescent | 0 | 0.0 | 0 | 0.0 | 2 | 15.4 |
| + Once a month | 0 | 0.0 | 0 | 0.0 | 2 | 15.4 |
| - Missing | 136 |  | 101 |  | 129 |  |
| Patient prefers ALN-EFF to any previous treatment for osteoporosis |  |  |  |  |  |  |
| - No previous treatment | 715 | 76.6 | 628 | 75.4 | 589 | 75.7 |
| - Yes | 198 | 21.2 | 190 | 22.8 | 181 | 23.3 |
| - No | 20 | 2.1 | 15 | 1.8 | 8 | 1.0 |
| + Reasons for not preferring (if freq > 10%) |  |  |  |  |  |  |
| + Not specified | 4 | 20.0 | 2 | 13.3 | 1 | 12.5 |
| + Posology | 4 | 20.0 | 4 | 26.7 | 3 | 37.5 |
| + Pharmaceutical form | 0 | 0.0 | 3 | 20.0 | 1 | 12.5 |
| + No preference | 0 | 0.0 | 2 | 13.3 | 1 | 12.5 |
| + Not easy | 0 | 0.0 | 0 | 0.0 | 1 | 12.5 |
| + She prefers Denosumab | 0 | 0.0 | 0 | 0.0 | 1 | 12.5 |
| - Missing | 10 |  | 9 |  | 7 |  |

* N stands for 'Patients on ALN-EFF'

ALN-EFF: buffered soluble alendronate 70 mg effervescent tablet
