# Supplementary material for: A Prospective Open‐Label Observational Study of a Buffered Soluble 70 mg Alendronate Effervescent Tablet on Upper Gastrointestinal Safety and Medication Errors: The GastroPASS Study
Source: JBMR Plus. 2021 May 17;5(7):e10510. doi: 10.1002/jbm4.10510 (PMC8260812; doi:10.1002/jbm4.10510)
Supplement: Supplementary file 6 — Supplemental Table S6. Discontinuation and Reasons for Discontinuation, by Follow‐up Time Window and at the End of the Study [file JBM4-5-e10510-s006.docx]

Supplementary Material S6. Discontinuation and reasons for discontinuation, by follow-up time window and at the end of the study.

|  | Early follow-up | | Intermediate follow-up | | Late follow-up | | Overall | |
| --- | --- | --- | --- | --- | --- | --- | --- | --- |
|  |  |  |  |  |  |  |  |  |
|  | n | % | n | % | n | % | n | % |
| N | 999 | 100 | 930 | 100 | 856 | 100 | 1028 | 100 |
| Patients discontinued permanently ALN-EFF | 53 | 5.3 | 85 | 9.1 | 71 | 8.3 | 209 | 20.3 |
| Reason for discontinuation |  |  |  |  |  |  |  |  |
| - Patient decision | 18 | 34 | 39 | 45.9 | 32 | 45.1 | 89 | 42.6 |
| - Lack of efficacy | 0 | 0.0 | 0 | 0.0 | 1 | 1.4 | 1 | 0.5 |
| - Tolerability / Adverse event | 38 | 71.7 | 45 | 52.9 | 15 | 21.1 | 98 | 46.9 |
| - Other* | 6 | 11.3 | 16 | 18.8 | 30 | 42.3 | 52 | 24.9 |
| - Dental care | 3 | 50.0 | 5 | 31.3 | 6 | 20.0 | 14 | 26.9 |
| - Medical advice | 0 | 0.0 | 1 | 6.3 | 6 | 20.0 | 7 | 13.5 |
| - Other concomitant conditions | 0 | 0.0 | 4 | 25.0 | 3 | 10.0 | 7 | 13.5 |
| - Patient condition | 0 | 0.0 | 2 | 12.5 | 2 | 6.7 | 4 | 7.7 |
| - Shortage | 0 | 0.0 | 0 | 0.0 | 11 | 36.7 | 11 | 21.2 |
| - Treatment issue | 0 | 0.0 | 1 | 6.3 | 0 | 0.0 | 1 | 1.9 |
| - Treatment change | 3 | 50.0 | 3 | 18.8 | 2 | 6.7 | 8 | 15.4 |

ALN-EFF: buffered soluble alendronate 70 mg effervescent tablet
